# Supplementary material for: Patterns of Intron Gain and Loss in Fungi
Source: PLoS Biol. 2004 Nov 30;2(12):e422. doi: 10.1371/journal.pbio.0020422 (PMC532390; doi:10.1371/journal.pbio.0020422)
Supplement: Table S1 — Also available at http://genes.mit.edu/NielsenEtAl/. (4.3 MB ZIP). [file pbio.0020422.st001.zip › NielsenEtAl/html/310.html]

AN1059.1.NCU01611.1.MG06981.1.FG00840.1


```
 CLUSTAL W (1.82) Multiple Sequence Alignments - Introns Inserted


Sequence 1: NCU01611.1	918 aa
Sequence 2: FG00840.1	854 aa
Sequence 3: MG06981.1	870 aa
Sequence 4: AN1059.1	804 aa
Alignment Length: 926 aa
Number Identitical Residues: 454 aa
Alignment Score (without introns) 21130


MG06981.1 	-MPSQVRIFTPPKPLGETLAEPDQDPVKAQQPLPRRQ------LKPMS----------KN
NCU01611.1	MTLTEVRTFTPPRAFGDAISQPVVTPVITTSTTPRSIPELQITLKPAISPSPKAR--QNS
FG00840.1 	--MTQVRTFTPPQSLGERLSDPSSAPTSAGIAVPRTLPDIPELAQPRRASPPRLSGDYKR
AN1059.1  	--MEAPRSHPAPEPK---IKPTTVDGGLTDQVKTKS------------------------
          	      * ...*..    :  .      :    .:                         

MG06981.1 	HGGIPEE---GGITFAAQDSLPKLPIPDLERTCEKYLAALKPLQSPREHGETRNAVQEFL
NCU01611.1	MASTPEEKSKGGVTFAHQDKLPKLPIPELESTCQRYLASLKPLQTHREHAETRRAVQEFL
FG00840.1 	YPMADESKKDGGITFAGQDKLPKLPIPELESSCKKYLEALKPLQTVREHAQTQHAVNEFL
AN1059.1  	----------GGITFAHQDSLPKLPIPDLESTCKKYLDALSPLQTPKEQEETKAAVREFL
          	          **:*** **.*******:** :*::** :*.***: :*: :*: **.***

MG06981.1 	RHDGPELQEKLKKYAEGQTSYIEQFW1YDSYLNFDNPVVLNLNPFFLLEDDPTPARNNQV
NCU01611.1	QTDGPELHEKLKKYAEDQTSYIEQFW1YDSYLNYDNPVVLNLNPFFLLEDDPTPARNNQV
FG00840.1 	KGDGPELHEKLKAYAEGKTSYIEQFW1YDSYLNFDNPVVLNLNPFFLLEDDPTPARNDQV
AN1059.1  	KGEGPGLQERLQKYATSQTSYIEQFW1YDSYLNYDSPVVLNLNPFFLLEDDPTPARNNQL
          	: :** *:*:*: ** .:******** ******:*.*********************:*:

MG06981.1 	TRAASLVVSALEFVRAVRKEELPPDKVKNTPLCMYQYSRLFGTARVPTENGCQIEQDPHS
NCU01611.1	TRAASLIASALEFVRAVRREELPPDTVRGTPLDMYQYSRLFGTARVPTDSGCQIQQDDKT
FG00840.1 	TRAASLVVSSLEFVRAVRKEELAPDKVKGTPLCMYQFSRLFGTARVPTEEGCQIEQDPES
AN1059.1  	TRAASLVVSALSFVRAVRREELPPDTVRGTPLCMYQYSRLFGTARVPTENGCVISQDPKS
          	******:.*:*.******:***.**.*:.*** ***:***********:.** *.** .:

MG06981.1 	KHIVVMAHGQFYWFDALDDNSDVIMTEKDISINLQTIVDDASLTPIQEAAKGALGVLSTE
NCU01611.1	KHIVVMCHGQFYWFDVLDDNSDAIMTEKDIAINLQTIVDDARQTPIQEAAKSALGVLSTE
FG00840.1 	KHIVVMCHGQFYWFDVLDDNSDVIMSEKDITINLQTIVDDATQVPIQDAAKGALGVLSTE
AN1059.1  	KHIVVMCRGQFYWFDVLDENHDQIMTEKDIAMNLEVILADAEQTPIQEAAKGALGVLSTE
          	******.:*******.**:* * **:****::**:.*: **  .***:***.********

MG06981.1 	NRKVWSGLRDILTRDEGSNNADCLGIIDSALFILCLDYTEPATAAELCQNMLCGTSEIEK
NCU01611.1	NRKVWSGLRDVLTSDPGSNNADCLSIVDSALFVVCLDYTEPTTAAALSQNMLCGTSEIEK
FG00840.1 	NRKVWSGLRDVLTSEPGSNNADSLGIVDTALFVLCLDYAEPTDAAALCQNMLCGTSEIEK
AN1059.1  	NRKVWSGLRDILTKDEHSINAEGLNIVDTALFVLCLDDTEPHNMSELCANMLCGTNVVEK
          	**********:** :  * **: *.*:*:***::*** :**   : *. ******. :**

MG06981.1 	GVQIGTCTNRWYDKLQIIVCKNGSAGINFEHTGVDGHTVLRFASDVYTDTILRFARTING
NCU01611.1	GVQIGTCTNRWYDKLQIIVCKNGSAGINFEHTGVDGHTVLRFASDIYTDTILRFARSING
FG00840.1 	GVQIGTCTNRWYDKLQIIVCKNGSAGINFEHTGVDGHTVLRFASDVYTDTILRFARTING
AN1059.1  	GVQVGTCTNRWYDKLQIIVCKNGSAGINFEHTGVDGHTVLRFASDVYTDTILRFARTING
          	***:*****************************************:**********:***

MG06981.1 	HSPTLWASTSPDPSKRDPESFGDVIPTPHKLEWDLVPEVSIAVRFAETRLADLIGQNEFE
NCU01611.1	KAPSMWASTSPDPSKRDPESFGDVSTTPHKLEWDMGPELSVAVRFAETRLADLIEQNEFQ
FG00840.1 	MAPSLWASSSPDPSKRDPESFGDVSVTPRKLEWDMIPELSVAVRFAETRLADLIEQNEFQ
AN1059.1  	QAPSLWATTSPDPSKRHPRSFGDVSPTPRKLEWDMLPELSIAIRFAESHLADLLQQHEFQ
          	 :*::**::*******.*.*****  **:*****: **:*:*:****::****: *:**:

MG06981.1 	CLDFSAYGKNFITSMGFSPDAFVQMAYQAAYYGLYGRAECTYEPAMTKFFLHGRTEAVRS
NCU01611.1	CLDFHGYGKNFITSMGFSPDAFVQMAFQAAYYGLYGRVECTYEPAMTKMFLHGRTEAIRT
FG00840.1 	TLDFSHYGKNFITSMGFSPDAFVQMAFQAAYYGLYGRVECTYEPAMTKMFLHGRTEAIRT
AN1059.1  	VLDFKGFGKNFITSMGFSPDAFVQMAFQAAYYGLYGQVVNTYEPAMTKAFLHGRTEAIRS
          	 ***  :*******************:*********:.  ******** ********:*:

MG06981.1 	VTDESINFIQNFWADNPAEAKVEALRAACKKHVNMTKDCAKAQGCDRHLYALFCLWQRLV
NCU01611.1	VSDDSVNFVQTFWADAPGDAKIDALRKACQAHVNKTRDCAKAQGCDRHLYAMLCLWQRMQ
FG00840.1 	VSEEVVDFVQTFWAENPAEAKIEALRKACQKHTANTRQCAKAEGCDRHLYALFSVWQRSL
AN1059.1  	VTNEALDFVKTFWAENPAEDKVNALRKATEKHSAITKECSKGQGQDRHLYALYTLWQRQF
          	*::: ::*::.***: *.: *::*** * : *   *::*:*.:* ******:  :***  

MG06981.1 	DEDAGTTTNGYSSGMSDNEGAVSERSPSPTATTATATGEKEKENGEGLRSRGESTSS---
NCU01611.1	DDDAASTT-TDNSMNG-YSFEDGSIIGSPSKDSVFSQSMESQSVNGGGRERGDSINSHIS
FG00840.1 	DEDLDYSN-GYSSPGDEYSDQTGSPVGSPVQGS--HPSIDGVEVR-PTRDRGYSVNS---
AN1059.1  	DEAPSSRN--NSVDEASNGYTSPAEIDSIAGSPLSPSSVSEVDGLSSTGSYGRAMRN---
          	*:     .   .    .          *    .    . .  .      . * :  .   

MG06981.1 	---SQRSVKYATLPLIFADPGWDKLNTTILSTSNCGNPSLRQFGFGPVSGDGFGIGYIIK
NCU01611.1	ATPSQRGGVPQPLPAIFADAGWDKLNTTILSTSNCGNPSLRHFGFGPVSGEGFGIGYIIK
FG00840.1 	-----RSRDQAPLPALFADAGWDKLNNTILSTSNCGNPALRQFGFGPTSGDGFGIGYIIK
AN1059.1  	---------LPPTPAIFSDPGWDKINNTILSTSNCGNPCLRHFGFGPTSADGFGIGYIIK
          	           . * :*:*.****:*.***********.**:*****.*.:*********

MG06981.1 	DERISMCVASKHRQTKRFVDALESYLLEIRRILRLASRRAASSTADGGPLAGSIAEAKQQ
NCU01611.1	DECISICVSSRHRQTRRFVDTLESYLLEIRRILRIAARNAASAGGVGATGT----TGNKG
FG00840.1 	DEGLAICVASKHRQTKRFVDTFESYLLEIRRILRIGNRKMSTG----------------K
AN1059.1  	DDSISICASSKHRQTARLMQTLELYLLEIRKLLRATVHKPSSP----------------R
          	*: :::*.:*:**** *:::::* ******::**   :. ::                  

MG06981.1 	TTRAREVDDEQRQQYRR----PSQQTNRLKSRGRMIAG----------------PGLEGL
NCU01611.1	MSRAREIDAAAINQNGQEKLARPKIGNRIKSRGRLITGQSSGLMGHGGPTSMYTNGGGSS
FG00840.1 	ASRAREVEIE-----------RPKLLSRIKSRGRPIT----------------------A
AN1059.1  	TSRAREMEMI------------AERVQRDQRRGRIVR-----------------------
          	 :****::              .:  .* : *** :                        

MG06981.1 	MRSKSSMTG--TTSPTSESLAMSEDDELGGY1GFFDAGMLLQALKARNT-----HFDVGE
NCU01611.1	VGNIKGAGGGSVSSAMEESVVMSEDDEIGGY1GFFDAGMLQQALKARQQAHYHHHSHNHD
FG00840.1 	VESLRSATG--TTSPTNESSTFSEDDEMGGY1GFFDAGMLLQALKARSE-----NFESSD
AN1059.1  	TDAGHLRAG--TETPTTDSGDMD-DDGMGGY1GFFDAGMLLHALKGLNVD----------
          	        *  . :.  :*  :. ** :*** ******** :***. .            

MG06981.1 	TRASER-AAVQARR--NNVGKKLRLIDY
NCU01611.1	SSSDDRSNQSRQRRSRRDVGKKLPLIDY
FG00840.1 	SRASERAAAAQARR--RDIGKKLRLSDY
AN1059.1  	---RERGGDKTPRR--RFVGKKLRLNEY
          	    :*.     **  . :**** * :*
```
